# Mobile Phone Apps for Food Allergies or Intolerances in App Stores: Systematic Search and Quality Assessment Using the Mobile App Rating Scale (MARS)

**DOI:** 10.2196/18339

**Published:** 2020-09-16

**Authors:** Floriana Mandracchia, Elisabet Llauradó, Lucia Tarro, Rosa Maria Valls, Rosa Solà

**Affiliations:** 1 Functional Nutrition, Oxidation, and Cardiovascular Diseases Group (NFOC-Salut), Healthy Environment Chair Facultat de Medicina i Ciències de la Salut Universitat Rovira i Virgili Reus Spain; 2 Unit of Nutrition and Health EURECAT-Technology Centre of Catalonia Reus Spain; 3 Hospital Universitari Sant Joan de Reus Reus Spain

**Keywords:** food allergy, food hypersensitivity, food intolerance, allergens, mobile applications, mobile health, mHealth, eHealth.

## Abstract

**Background:**

Food allergies and intolerances are increasing worldwide, and mobile phone apps could be a promising tool for self-management of these issues.

**Objective:**

This study aimed to systemically search and assess food allergy or intolerance apps in app stores using the multidimensional Mobile App Rating Scale (MARS) to rate the objective and subjective quality and to identify critical points for future improvements.

**Methods:**

This systematic search identified apps through the keywords “food allergy,” “food intolerance,” and “allergens” in English, Spanish, and Italian in the Apple App Store (iOS) and Google Play Store (Android). The inclusion criteria were a user star rating of ≥3 (of 5 stars) to limit the selection to the most highly rated apps; ≥1000 reviews as an indicator of reliability; and the most recent update performed up to 2017. Then, the apps were divided according to their purpose (searching for allergen-free “food products,” “restaurants,” or recipes in “meal planners”) and evaluated on a scale of 1 to 5 points using the MARS in terms of (1) app classification category with a descriptive aim; (2) app subjective and objective quality categories comprised of engagement, functionality, esthetics, and information sections (Medline was searched for eligible apps to check whether they had been tested in trials); and (3) an optional app-specific section. Furthermore, the output and input features were evaluated. Differences between MARS sections and between app purposes and correlations among MARS sections, star ratings, and numbers of reviews were evaluated.

**Results:**

Of the 1376 apps identified, 14 were included: 12 related to food allergies and intolerances that detect 2-16 food allergens and 2 related only to gluten intolerance. The mean (SD) MARS scores (maximum 5 points) were 3.8 (SD 0.4) for objective quality, highlighting whether any app had been tested in trials; 3.5 (SD 0.6) for subjective quality; and 3.6 (SD 0.7) for the app-specific section. Therefore, a rating ≥3 points indicated overall acceptable quality. From the between-section comparison, engagement (mean 3.5, SD 0.6) obtained significantly lower scores than functionality (mean 4.1, SD 0.6), esthetics (mean 4, SD 0.5), and information (mean 3.8, SD 0.4). However, when the apps were compared by purpose, critical points were identified: meal planner apps showed significantly higher engagement (mean 4.1, SD 0.4) than food product (mean 3.0, SD 0.6; *P*=.05) and restaurant (mean 3.2, SD 0.3; *P*=.02) apps.

**Conclusions:**

In this systematic search of food allergy or intolerance apps, acceptable MARS quality was identified, although the engagement section for food product and restaurant purpose apps should be improved and the included apps should be tested in trials. The critical points identified in this systematic search can help improve the innovativeness and applicability of future food allergy and intolerance apps.

## Introduction

Food allergies and intolerances are adverse reactions to the ingestion of, contact with, or inhalation of a specific food, derivative, or additive [1]. The prevalence of such adverse food allergy and intolerance reactions is increasing worldwide, especially in developed countries [2].

On the one hand, food allergies involve an immune-mediated reaction that occurs between a few minutes and 1 hour after exposure to the allergen, with symptoms ranging from moderate to severe [3]. The prevalence of food allergies is higher in children (<10%) than in adults (approximately 1%-2%) [3]. On the other hand, food intolerances are nonimmunological hypersensitivity responses due to a nontolerated dose of a food or a component of a food, with symptoms or signs occurring several hours after food consumption and lasting from hours until several days afterward [4]. Food intolerances are more common worldwide than food allergies, affecting up to 15%-20% of the general population [5].

Although new approaches to food allergies have recently been under clinical investigation [6], one strategy is to correctly identify food allergens to avoid the consumption of even small amounts of an allergen that causes a reaction [7]. To help consumers easily identify food allergens in food products, prepackaged or not, European legislation from 2014 (EU Food Information for Consumer Regulation No. 1169/2011) requires food businesses to clearly provide consumers, through labels or other verbal or written communications, with information about nutritional values and the presence of any of 14 specified food allergens (cereals containing gluten, crustaceans, eggs, fish, peanuts, soya, milk, nuts, celery, mustard, sesame, sulfur dioxide, lupin, and mollusks) [8]. Despite the European legislation, a 2019 study showed gaps in compliance with the regulation, finding that only 83 of the 295 evaluated restaurants (28.1%) labeled food allergens on the menus and that the restaurant staff had deficiencies in their food allergen knowledge and management [9]. In addition to relying on the information provided by food businesses and their employees, consumers must fundamentally self-monitor and self-manage their health [10].

In this context, there is increasing interest in mobile technology, such as apps, that focuses on helping consumers supervise what they are eating [11] by detecting allergens [12] not derived from cross-contamination and delivering specific health information [13,14] in relation to preparing daily meals, purchasing suitable food products, or searching for restaurants with allergen-free menus.

In recent years, mobile health (mHealth) technologies, including software, sensors, and mobile phones [15], have improved the management of health care services [16] and interventions such as the achievement of weight loss and smoking cessation as well as the management of several chronic and mental diseases [17]. Currently, the potential of apps for food-related conditions [18] such as food allergies and intolerances, whose incidence is growing worldwide [19], is also being studied. The convenience of apps in health management is favored by approximately 59% of the world population, corresponding to 4.57 billion people, mostly in northern Europe and the United States, who were active internet users in 2020 [20]. Apps enabling consumers to identify food allergens in foods and products, find allergen-free restaurants, and report and evaluate symptoms related to food allergies are already available, but most of them offer irrelevant and poor content [21].

Since plenty of apps currently exist, their reliability must be verified [22], as the traditional systems used to test app quality, such as users’ star ratings (evaluating apps on a scale of 1 to 5 stars) and reviews, could allow fake or subjective reviews, giving wrong indications to users [23]. Furthermore, app descriptions in app stores are often incomplete or incorrect and are not a valid tool for assessing the quality of an app [24], especially when dealing with sensitive topics such as food allergies.

The necessity of regulating the quality and safety of mHealth technologies, defined by the World Health Organization as medical and public health practices supported by mobile phones, patient monitoring devices, personal digital assistants, and other wireless devices [25], is particularly important for apps intended to be used for the diagnosis, cure, mitigation, treatment, and prevention of a disease or other conditions by aiding clinical decision-making [26]. These kinds of apps are classified and regulated as medical devices by the US Food and Drug Administration to ensure the safety of apps that are recommended by health professionals to their patients [26]. For instance, in 2015, the government of Catalonia (Spain) introduced a public platform for apps with quality accreditation from health professionals (mConnecta platform), thus establishing a safe and reliable environment for people to use these mHealth apps to self-monitor their health practices [27]. However, nonmedical apps intended to provide information and education to users, such as apps for food allergies and intolerances, also need to be regulated since incomplete information is often provided [28]. In this way, apps will provide better information to help users make health-related choices [29], mHealth will have more value, and fewer ineffective and unsafe apps will be available [30].

Owing to the necessity of ensuring better app quality for users, a Mobile App Rating Scale (MARS) was developed by a multidisciplinary team of experts as a simple, objective, and reliable tool for researchers, developers, and health professionals to assess app quality and provide suggestions for future designs [31]. The MARS tool provides a multidimensional evaluation of app quality, whereas other existing tools mostly use one-dimensional measures. For example, the Intercontinental Medical Statistics Institute for Healthcare Informatics tool [32] assesses only app functionality, and the criteria of the Health Care Information and Management Systems Society tool [33] evaluate only app usability. The MARS tool has already been used for the quality assessment of different apps related to nutrition [34-36], sleep management [37], food provision [38], calorie counting [39], smoking cessation [40], physical activity [41], and weight management [42] but has not previously been used for food allergy or intolerance apps.

The aim of this paper was to systemically search app stores for apps about food allergies or intolerances, to assess the apps using the multidimensional MARS ratings of objective and subjective quality, and to identify the critical points for future improvements of these apps.

## Methods

### Search Strategy

The present study featured a systematic search and content analysis of apps about food allergies or intolerances available in the Apple App Store (iOS) and Google Play Store (Android). The apps were searched by the two authors between May 2019 and June 2019. The searches were conducted anonymously by logging out of the user accounts for the stores. Specific keywords such as “food allergy,” “food intolerance,” and “allergens” in English, Spanish, and Italian were used to search for the available apps in any of these 3 languages.

### App Selection

**Figure 1:**
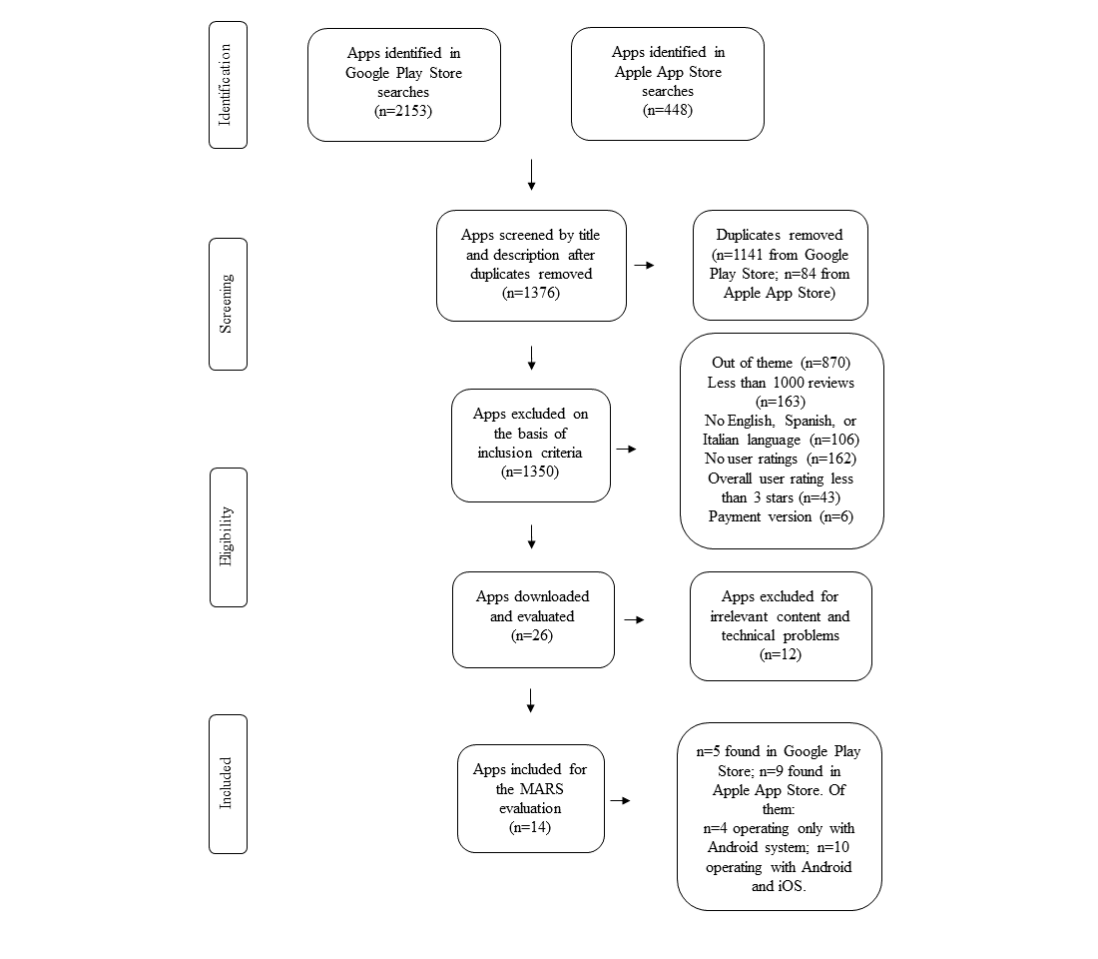
Flow diagram for the selection process of the apps included in the study.

The app selection process is described in Figure 1. Specific inclusion and exclusion criteria were applied to limit the search to the most relevant and reliable apps, in line with previous studies [35,38,41,42]. In particular, only apps that offered a free version were included in the search, as they are most commonly used by the general population. Apps in English, Spanish, and Italian were considered if they had (1) a minimum user star rating ≥3 (of 5 stars) to limit the search to the apps most highly rated by users, (2) ≥1000 reviews to identify the apps that were most commonly used and experienced, and (3) a last update up to 2017 to evaluate the most recently produced and revised apps. Finally, apps were included if their aim was to help allergic or intolerant users select suitable food products to buy or consume, personalize their daily nutrition on the basis of their needs and food restrictions, detect allergens in recipes and food product labels, search for specific restaurants or supermarkets according to their needs, and obtain information and advice about allergen self-management. Duplicates and apps that did not fulfill the aforementioned inclusion criteria or did not work were excluded from the study.

### Data Extraction

All the identified apps were registered in an initial list to count the total number of apps and the number of duplicates. The general characteristics of the included apps were extracted from the information in the app stores, while the main app features were verified by the authors by using the app. Furthermore, the features were categorized as input and output features on the basis of whether the app content was created by the users or automatically generated.

After data extraction, the authors divided the apps according to 3 purpose types (considering that the apps included presented different purposes): (1) searching for allergen-free food products, (2) searching for restaurants offering menus adapted to different food allergies and intolerances, and (3) functioning as meal planners for suitable daily meals according to users’ food allergies or intolerances. This division of the included apps allowed us to compare the MARS quality ratings among apps with a similar purpose and to provide suggestions for future app designs in line with this purpose.

Moreover, web-based searches on the Medline database were conducted by app name (Eat This Much, Fitberry, Mealime, Recetas Vegetarianas y Veganas, SideChef, Tasty, Mercadona, Mi Intolerancia Alimentaria, Open Food Facts, ¿Qué Puedo Comer?, Club VIPS, Find Me Gluten Free, Foster’s Hollywood, and Happy Cow) and by “apps for food allergies and/or intolerances” to determine whether they had already been evaluated in scientific trials.

### MARS App Quality Assessment

App quality was assessed using the MARS rating scale, a reliable tool with a high internal consistency (α=0.90) and an interrater reliability interclass correlation coefficient of 0.79 [31]. The following steps were taken. First, before assessing the app quality, the authors followed specific web-based training organized by the MARS developers [43], such as an exercise to better understand how to classify the apps. Then, to experience and test the functionality of the included apps, the two authors independently used each of the 14 apps for 1 month. Finally, the quality assessment was conducted in agreement between the two authors, and disagreements were resolved through discussion with a third author.

The MARS rating scale consists of 2 categories. The first is the app classification category, with 6 items of descriptive and technical information for each app: (1) descriptive information (name, number, and type of ratings for all versions; developer; version; cost; platform; description; update), (2) focus, (3) theoretical background and strategies, (4) affiliations, (5) age group, and (6) technical aspects (login, password protection, web access, app community, social sharing, and reminder functions). The second category is the app quality category, which is divided into objective and subjective quality. Objective quality has 4 sections (engagement, functionality, esthetics, and information) with 19 items, while subjective quality is comprised of 4 items, for a total of 23 items.

In addition to these 2 categories, there is an optional app-specific section with 6 items to collect further information about the perceived impact of the app on the user (awareness, knowledge, attitudes, intention to change, help-seeking, behavior change).

The app classification category was not rated since its purpose was only descriptive. Instead, to evaluate the app quality category, each item was scored on a 5-point rating scale from 1 to 5 (1: inadequate; 2: poor; 3: acceptable; 4: good; 5: excellent). For each app, the total mean score was the sum of the score of each item divided by the number of total items. The mean score of the 4 objective quality sections (engagement + functionality + esthetics + information) was calculated separately from that of the subjective and app-specific sections to strengthen the impartiality of the measure.

For each objective quality section, the maximum score was 25 points for engagement, 20 points for functionality, 15 points for esthetics, and 35 points for information, for a total of 95 points for objective quality. Subjective quality could reach a maximum of 20 points, and the app-specific section could reach a maximum of 30 points.

In addition to the objective and subjective quality ratings, the app-specific section was evaluated on the 5-point rating scale.

### Statistical Analysis

Continuous variables of the scores obtained for each section of the MARS quality assessment, with the exception of the app classification category, are presented as the mean and SD. Categorical variables for the included apps and their input and output features are presented as percentages. Multiple comparisons between the 3 purposes of the included apps (food products, restaurants, meal planners), MARS scores, and user star ratings were performed and adjusted using the generalized linear model of the Bonferroni test. Correlations among the MARS scores, user star ratings, and number of reviews were analyzed using Pearson correlation coefficients (for normally distributed variables) and Spearman correlation coefficients (for not normally distributed variables), which were interpreted as strong or moderate according to previously published cutoff points [44]. The analysis was performed with SPSS Statistics version 25. Statistical significance was considered at *P*≤.05.

## Results

### App Selection

Figure 1 shows the flowchart of the app selection process. After the removal of duplicates found in both stores, 1376 apps about food allergies or intolerances were screened by title and description by the two authors, resulting in 1350 apps being excluded on the basis of the inclusion criteria. To further evaluate the eligibility of their content, 26 apps were downloaded, and 12 of these were excluded by common agreement because of irrelevant content (apps from the same developer with equivalent features and findings) and technical problems. As a result, 14 apps about food allergies or intolerances were finally included in the study for quality assessment using the MARS tool; 5 of the 14 (36%) were found in the Google Play Store, and 9 of the 14 (64%) were found in the Apple App Store. Moreover, 4 of the 14 apps (29%) operated only on the Android system, and 10 of the 14 apps (71%) operated on both the Android and iOS systems. None of the included apps had previously been evaluated in scientific trials.

### Data Extraction: App General Characteristics

The general characteristics of the 14 included apps about food allergies or intolerances, shown in Multimedia Appendix 1*,* are described in the following sections.

#### App Purpose

First, the 14 included apps were divided according to their purpose.

Of the 14 apps, 6 (43%) were meal planners, helping users search for and plan meals adapted to allergies or intolerances. In particular, 4 apps (Tasty, Recetas Vegetarianas y Veganas, SideChef, and Fitberry) propose food recipes that can be filtered by the users’ allergies or intolerances and on the basis of personal preferences, such as cooking difficulty and type of meal, diet, and cuisine. The other 2 apps (Mealime and Eat This Much) are meal planners that allow weekly meals to be organized on the basis of personal preferences, dietary goals, and food restrictions, such as food allergies and intolerances. In this way, users can create a personal profile indicating allergens to eliminate from their diet and organize their daily or weekly diet plan, choosing among the dishes proposed automatically by the apps and filtering them by the selected allergen.

Of the 14 apps, 4 (29%) function as food product search tools, helping users search for suitable food products according to their food allergies and/or intolerances. In particular, 3 apps (Open Food facts, ¿Qué Puedo Comer?, and Mercadona) help users search, through barcode scanning or database searches, for the most suitable food by showing the allergens declared on the food product label and indicating the nearest place to buy them, and 1 app (Mi Intolerancia Alimentaria) is a calculator of food compatibility. According to the presence of an allergen, the user’s individual tolerance of the food or meal is calculated and shown using a 3-color code alert system (red, orange, and green) according to whether the compatibility of the food is low, medium, or high.

Restaurant searches represented the main purpose of 4 of the 14 apps (29%), helping users search for restaurants that offer menus adapted for allergic or intolerant consumers. In particular, 1 app (Happy Cow) searches for gluten-free, vegetarian, and vegan restaurants, hotels, supermarkets, and caterers; 1 app (Foster’s Hollywood) belongs to a popular restaurant chain and offers the possibility of looking at the restaurant’s allergen-free menu by checking the available meals in advance; 1 app (Club VIPS) searches for the nearest locations of different restaurant chains with allergen-free options; and 1 app (Find Me Gluten Free) searches for restaurants with gluten-free options.

#### Operating System

Of the 14 apps, 10 (72%) operate on both the Android and iOS systems, and 4 (28%) operate only on the Android system.

#### Number of Reviews

The number of reviews of the included apps varied from 1013 to 48,597 reviews.

#### Languages Available

Of the 14 apps, 4 (28%) are available only in Spanish, 5 (36%) are available only in English, and 5 (36%) are offered in 3-130 different languages.

#### Actions

The included apps enable users to benefit from different actions for the daily management of food allergies or intolerances.

#### Focus

Of the 14 apps, 12 (86%) are related to food allergies or intolerances, while 2 (14%) deal with gluten intolerance only.

#### Allergens Detected

The included apps differed in the number of allergens detected. Specifically, 10 of the 14 apps for food allergies identified milk and eggs; 9 identified crustaceans, peanuts, and nuts; 8 identified fish and soya; 6 identified sesame, mustard, and sulfur dioxide; 5 identified celery and lupin; and 1 identified wheat and grain. In addition, all 14 of the apps for food intolerances identified gluten, 4 identified lactose, and 2 identified fructose, sorbitol, histamine, and salicylic acid.

Thus, 5 of the 14 apps (36%) detected all 14 allergens that must be declared in the European Union (cereals containing gluten, crustaceans, eggs, fish, peanuts, soya, milk, nuts, celery, mustard, sesame, sulfur dioxide, lupin, and mollusks) above other food allergens present on the food product label, 2 of the 14 apps (14%) detected only gluten, and 7 of the 14 apps (50%) detected 2-10 food allergens.

#### Input and Output Features

The app features were distinguished as output features (Multimedia Appendix 2), where content is automatically generated by the app, and input features (Multimedia Appendix 3), where content is inserted and created by the user.

The lowest-rated app in the objective quality category, Mi Intolerancia Alimentaria (mean MARS score 3.2, SD 0.5), has fewer output features (4 of the 20 features) than the apps scoring >4 points, which offer 12-15 of the 20 output features and also had the highest scores in the engagement section. The same situation occurred for the input features, with apps scoring >4 points offering 8-9 of the 9 input features.

According to the app purposes, the most used features for the meal planner apps were allergen detection, search filters, sending of reminders and notifications, shopping list creation, suggestions and tips, rating and reviewing possibilities, personal profile, creation of a favorites list, and social sharing. For the food product apps, the most used features were allergen detection, listing of ingredients and additives, personal profile, and rating and reviewing possibilities. For the restaurant apps, the most used features were allergen detection, search filters, prompts and discounts, geolocation, rating and reviewing possibilities, personal profile, and social sharing.

### MARS App Quality Assessment

The MARS app classification category is the part that collects descriptive and technical information about the included apps. Descriptive data include general information (app name, rating of all versions, developer, number of ratings of all versions, version, cost of basic and upgraded versions, platform, description and last update, focus, theoretical background and strategies, affiliations, age group) and technical aspects present in the app description in the app store. Of these data, only the relevant aspects were extracted (focus, theoretical strategies, affiliation, age group, and technical aspects); they are described in Multimedia Appendix 4.

According to the MARS evaluation, the quality of the 14 included apps assessed in terms of the 4 objectives (engagement, functionality, esthetics, and information) and the one subjective section are shown in Multimedia Appendix 4*.* Additionally, the results of the optional app-specific section are included.

The overall mean (SD) MARS objective quality score, which allows the evaluation of the general app quality (maximum of 5 points), was 3.8 points (SD 0.4 points); thus, the quality of the 14 included apps was considered acceptable. The score of the subjective quality section was 3.5 points (SD 0.6 points), and that of the app-specific section was 3.6 points (SD 0.7 points).

In particular, the mean scores of the 4 single objective quality sections, from the highest to the lowest score, were as follows: functionality section, 4.1 points (SD 0.6 points); esthetics section, 4 points (SD 0.5 points); information section, 3.8 points (SD 0.4 points); and engagement section, 3.5 points (SD 0.6 points).

When the scores of the 6 MARS sections (4 objective, 1 subjective, and 1 app-specific) were compared, the score of the esthetics section (mean 4, SD 0.5) was significantly higher than that of the engagement section (mean 3.5, SD 0.6; *P*=.007), and the score of the functionality section (mean 4.1, SD 0.6) was significantly higher than that of the subjective quality section (mean 3.5, SD 0.6; *P*<.001). Moreover, the score of the information section (mean 3.8, SD 0.4) was significantly higher than that of the subjective quality (mean 3.5, SD 0.6; *P=*.002) and app-specific (mean 3.6, SD 0.7; *P=*.001) sections. No further significance was found in the other between-section comparisons.

Among the 3 app purposes (food products, restaurants, and meal planners), comparisons between the MARS sections, as shown in Table 1, were evaluated. The score of the engagement section was significantly higher for meal planner apps (mean 4.1, SD 0.4) than for the food product (mean 3.0, SD 0.6; *P*=.05) and restaurant (mean 3.2, SD 0.3; *P*=.02) apps. Furthermore, it emerged that for meal planner apps, the scores of the engagement (mean 4.1, SD 0.4; *P*=.04) and functionality (mean 4.3, SD 0.7; *P*=.02) sections were significantly higher than those of the subjective quality section (mean 3.9, SD 0.5), and the score of the functionality section was significantly higher than that of the esthetics section (mean 4.3, SD 0.3; *P*=.04). No further significance was found in the other between-section comparisons among the 3 app purposes.

**Table 1:** Differences in the mean MARS scores between app purposes.

Mean MARS^a^ scores	Meal planners	Food products	Restaurants	*P* value^b^	*P* value^c^	*P* value^d^
Engagement	4.10	3.00	3.20	.05	.02	1.0
Functionality	4.29	4.12	3.69	1.0	.43	.96
Esthetics	4.28	3.67	3.83	.17	.46	1.0
Information	3.97	3.79	3.62	1.0	.79	1.0
Subjective quality	3.87	3.31	3.19	.46	.26	1.0
App-specific	3.97	3.46	3.25	.75	.35	1.0

^a^MARS: Mobile App Rating Scale.

^b^Comparison between meal planners and food products.

^c^Comparison between meal planners and restaurants.

^d^Comparison between food products and restaurants.

### Additional Analysis

The relationships between MARS score quality and user star rating and number of reviews were determined using correlations (described in Table 2) and showed that the star ratings were significantly and strongly positively correlated with the MARS engagement section (r=0.69; *P*=.007) and app-specific section (ρ=0.79; *P*=.001). A moderate correlation was also found between MARS subjective (r=0.63; *P*=.01) and total objective quality (r=0.60; *P*=.02). However, no significant correlations were found between MARS sections and number of reviews.

**Table 2:** Correlation coefficients between MARS scores, user star ratings, and number of reviews.

Mobile App Rating Scale (MARS)	Number of reviews	Star ratings	*P* value^a^	*P* value^b^
Functionality^c^	0.13	0.33	.65	.25
Esthetics^c^	0.11	0.43	.71	.12
App-specific^c^	0.05	0.79	.87	.001
Number of reviews^c^	1.00	0.30	N/A^d^	.30
Engagement^e^	0.20	0.69	.50	.007
Information^e^	–0.14	0.42	.62	.14
Total objective quality^e^	0.03	0.60	.92	.02
Subjective quality^e^	–0.03	0.63	.93	.01
Star ratings^e^	0.30	1.00	.29	N/A

^a^Correlation between MARS scores and number of reviews.

^b^Correlation between MARS scores and star ratings.

^c^Spearman (*ρ*).

^d^N/A: not applicable.

^e^Pearson (*r*).

In addition, to verify whether the star ratings assessed by users were similar to the MARS scores obtained in our study, the comparisons were analyzed. The star ratings were significantly higher (mean 4.2, SD 0.4) than the MARS subjective quality score (mean 3.5, SD 0.6; *P*=.04).

## Discussion

The present systematic search and quality assessment study provides information about the objective (engagement, functionality, esthetics, and information) and subjective quality of the available apps for food allergies or intolerances in app stores. The quality assessment using the MARS tool indicated that the overall app quality of the 14 included apps was acceptable, according to MARS mean ratings of ≥3 from a maximum of 5 points.

By comparing the 6 MARS sections (4 objective quality, 1 subjective quality, and 1 app-specific), the most significant results were related to the apps’ functionality, esthetics, and information, as they appeared visually pleasant, sufficiently descriptive, well arranged, and easy to use, whereas the engagement section of most of these apps needs to be improved. As observed in other studies, apps with simple functionality can motivate people who have no familiarity with technology to adopt mobile apps [45]. Moreover, esthetics, such as visual attractiveness, is another key element for increasing users’ motivation to use the app [46].

Regarding the information section, the included 14 apps clearly presented their content through the support of images, graphics, and videos. Nevertheless, none of the apps has been tested in scientific trials, which is an important aspect of this section of the MARS tool. In addition, it is important to evaluate the apps’ efficacy in helping consumers self-manage food allergies or intolerances, since previous studies have demonstrated that commercial apps do not always provide the expected results when they are evaluated in trials [47-49]. For meal planner apps, future trials could evaluate the improvement in user knowledge and awareness of food allergens, which are considered important targets for the management of food allergies [50].

Moreover, the efficiency of food product apps should be tested in clinical trials to increase users’ confidence when food shopping and reading product labels. For the allergic and intolerant population, it is fundamental for the food labeling system to be available and comprehensive [51], and this kind of app could help consumers more quickly detect allergens in food products. Finally, for restaurant apps, customer satisfaction when eating away from home could be evaluated as a measure of food businesses’ compliance with the European regulation and with the allergen-free menus published on the app. Positive experiences when eating away from home are correlated with the availability of food allergen information provided by the restaurants [52].

Moreover, none of the 14 included apps claims any validation of the content by health professionals or allows remote support. Actually, a critical assessment published in 2015 found that most apps about food allergies lack important health information and are not developed with the support of health professionals [21]. It is important for such apps to be evaluated by health professionals to provide better information to help users make health-related choices [53-55]. Furthermore, apps providing professionally oriented support and communication are more engaging and favored by users, especially adolescents [54].

The results obtained in the present study indicate that app engagement is the section with the lowest score with respect to functionality, esthetics, and information, in line with other MARS assessments of apps for food provision [38], checking for drug interactions [56], and drunk driving prevention [57], and the lack of interactive features influences the engagement quality of these apps. However, in a comparison of the 3 purposes of the included apps, the engagement section of the meal planner apps received higher quality scores than that of the food product and restaurant apps. In fact, food product and restaurant apps do not use interactive features that motivate users to use them repeatedly [58], but for these apps, which are designed for short and specific use such as finding restaurants or products, user engagement and daily use are not really as essential as in meal planner apps. However, including features such as tips and suggestions to support consumers’ decisions or sending notifications [59] to notify users of new products or restaurants could improve the user app experience, growth of the app community, and app competitiveness. To increase user enjoyment and participation, meal planner, food product, and restaurant apps should perhaps include features such as rewards, goal-setting options, challenges, and leader boards, which have been recognized as effective tools in past studies [60-62], especially in adolescent populations, where game competition can motivate users to participate [63]. Finally, features such as feedback and self-monitoring, which have been demonstrated in previous studies to be effective in increasing users’ motivation [32,58] and health behavior [64,65], should be available in apps focused on self-managing food allergies or intolerances; however, only 2 of the 14 apps included in the present systematic search offer these features.

The subjective quality and app-specific sections need to be improved in relation to the 3 purposes of the included apps (meal planners, food products, and restaurants). These sections refer to general users’ impressions of the app, which, if positive, would lead them to recommend and use it. In this context, the lack of enough engagement could influence users’ perceptions. Thus, it is important to increase users’ subjective quality perception and impact of the apps (app-specific) by reinforcing, for example, the engagement profile, as discussed earlier, which mainly influences users’ view of the app.

Based on the number of input and output features offered, among the meal planner apps, Eat This Much, Mealime, and SideChef were found to be the most practical for users, obtaining higher scores in the MARS assessment than other apps with the same purpose. Previous studies have shown that food allergies and intolerances impact people’s quality of life and emotional status, increasing anxiety and depression [66,67]. The avoidance of food allergens requires constant attention because their presence in food is not always evident or is unknown [68]. This problem becomes even more complicated when consumers have to adapt food recipes or make appropriate ingredient substitutions according to their allergy or intolerance [69] without accurate recommendations or support. In this sense, these 3 apps could better help users while providing suggestions for self-managing food allergies or intolerances in terms of cooking and daily menus. Among the food product apps, the ¿Qué Puedo Comer? app was the most practical for users, offering more features and gaining higher scores in the MARS assessment. This app helps consumers understand food product labels, detect food allergens, and search for food products according to allergies, intolerances, or dietary requirements. Since food product ingredients change regularly and consumers may need to read packaging labels several times [69], these apps can provide instant information and support [70]. Among the restaurant apps, the Find Me Gluten Free and Happy Cow apps were the most practical for users compared to others with the same purpose. The provision of food allergen information on restaurant menus is very important for consumers, and these kinds of apps encourage the dissemination of such information by making it easier to search for restaurants with allergen-free menus [52].

The correlations of star ratings with the app-specific, engagement, and subjective sections suggest that when evaluating an app, users refer more to the subjective impression of the app given by the engaging features offered than to the quality and quantity of the information provided [71], as shown by the results obtained in the present study. As observed in previous studies, there is an evident difference between the quality evaluation obtained by a researcher using a more objective tool such as the MARS and a real-world user who tends to evaluate app quality through star ratings in a much more subjective way [72]. Nevertheless, app store user star ratings cannot be totally trusted since they are sometimes derived from piloted reviews or paid bots deployed by the developer [73].

Thus, according to the results obtained, we consider that MARS quality assessment is a valid tool for providing more accurate app quality information and suggestions for future apps.

### Suggestions for Future App Development

Based on the present app assessment, several suggestions emerged for the future design of high-quality apps focused on improving the wellbeing of subjects with food allergies or intolerances:

1. Further features should be included, especially in meal planner apps, to improve the user app experience and increase participation.
2. Content should be validated by health professionals and scientists to provide users with more reliable information about food allergies or intolerances [36].
3. Remote support by health professionals would help users manage their food allergies or intolerances [54].
4. Testing in scientific trials would demonstrate the apps’ reliability and effectiveness [74] in detecting food allergens and improving user knowledge.
5. Regulation of nonmedical apps should be considered in the future since it would avoid the development of unrealistic and ineffective apps, provide more correct information to users [29], and provide more value to mHealth technology [30].
6. App quality should be evaluated through innovative methods, including multiple dimension perspectives, as in the MARS tool. The MARS tool, compared to other scales [32,33], represents a multidimensional evaluation of app subjective quality as well as engagement, functionality, esthetics, and information as indicators of objective quality. However, although the MARS tool has been widely tested, it should be validated in the near future [75] to increase its value, and, depending on the area of interest of the app (eg, health care, nutrition, sports, psychology), the items in each section should be more specific and theme-based. Apps for food allergies or intolerances, for example, should include items asking whether food allergen information is effectively and appropriately provided to users.

### Limitations

The present study also has several limitations. First, the majority of the apps about food allergies or intolerances found in the app stores had fewer than 1000 reviews and a user star rating <3, indicating low interest by users. Consequently, it was not possible to include most of the apps because we considered a rating of 3 stars as the minimum threshold for app quality. However, it was important for the inclusion criteria to limit the findings to the most reliable and popular apps, as the market includes plenty of dubious apps. Second, apps with only a paid version were excluded from the search. Third, several apps were excluded because of technical problems, such as being unable to open or use the app. Fourth, because this study is not a systematic review of the literature but is a systematic search of app stores, it was not possible to register it in PROSPERO [76]. Finally, despite the increasing attention to apps, the literature about the assessment of app quality is very scarce [77] and not oriented to food allergies and intolerances.

### Conclusions

In this systematic search of food allergy or intolerance apps, acceptable MARS quality was identified, although the engagement of food product and restaurant apps should be improved and the included apps should be tested in trials. The critical points identified in this systematic search can help improve the innovativeness and applicability of future food allergy and intolerance apps.

## Appendix

Multimedia Appendix 1 General characteristics of the mobile phone apps included in the review.

Multimedia Appendix 2 Output features of the included apps.

Multimedia Appendix 3 Input features of the included apps.

Multimedia Appendix 4 Mobile App Rating Scale (MARS) and user star ratings of the included apps in mean (SD).

## Abbreviations

IMS: Intercontinental Medical Statistics
MARS: Mobile App Rating Scale
mHealth: mobile health
