# Supplementary material for: Mobile Phone Apps for Food Allergies or Intolerances in App Stores: Systematic Search and Quality Assessment Using the Mobile App Rating Scale (MARS)
Source: JMIR Mhealth Uhealth. 2020 Sep 16;8(9):e18339. doi: 10.2196/18339 (PMC7527917; doi:10.2196/18339)
Supplement: Multimedia Appendix 1 [file mhealth_v8i9e18339_app1.pdf]

App name	App purpose	Operating system	N° reviews	Languages available	Actions	Focus		Allergens detected
						Allergy	Intolerance	
1. Eat This Much	Meal planners	Android, iOS	3,863	English	1. Set your food preferences: diet type, foods to exclude or limit (sodium, cholesterol) and allergen-free diet options;<br>2. Plan your meals for the week;<br>3. Set weight goals;<br>4. Make your grocery shop list;<br>5. Cook with step-by-step instructions;<br>6. Look at the nutritional info of your meal;<br>7. Integrate Apple Health app.	x	x	Gluten, milk, crustaceans, fish, peanuts, nuts, soya and eggs (8 food allergens in total).
2. Fitberry	Meal planners	Android, iOS	1,064	English, +6	1. Make your personal profile and set your allergies/intolerances and preferences (diet type, nutrition goals, allergen-free diet);<br>2. Search for recipes, ingredients and meal plans;<br>3. Cook with step-by-step instructions;<br>4. Plan your meals for the week;<br>5. Get a personalized weekly magazine.	x	x	Gluten, milk, crustaceans, peanuts, nuts, eggs, sulfur dioxide, wheat and grain (9 food allergens in total).
3. Mealime	Meal planners	Android, iOS	11,213	English	1. Make your personal profile and set your food restrictions and preferences;<br>2. Plan your meals for the week;<br>3. Make your grocery shop list;<br>4. Cook with step-by-step instructions;<br>5. Integrate Apple Health app.	x	x	Gluten, milk, crustaceans, fish, peanuts, nuts, soya, eggs, sesame and mustard (10 food allergens in total).
4. Recetas Vegetarianas y Veganas	Meal planners	Android	2,743	Spanish	1. Search for vegetarian and vegan recipes according to your preferences (the type of diet, meal, allergy/intolerance, cooking time, nutritional values, chef);<br>2. Cook with step-by-step instructions and look at the nutritional values;<br>3. Make your grocery shop list;<br>4. Find suggestions and tips.	x	x	Gluten, lactose and eggs (3 food allergens in total).
5. SideChef	Meal planners	Android, iOS	3,950	English	1. Make your personal profile and set your allergies/intolerances, preferences and cooking goals;<br>2. Plan your meals for the week;<br>3. Make your grocery shop list and buy online;<br>4. Cook with step-by-step instructions<br>and control your	x	x	Gluten, milk, crustaceans, fish, peanuts, nuts, soya and eggs (8 food allergens in total).
6. Tasty	Meal planners	Android, iOS	48,597	English	1. Select type of meal, cooking difficulty, meal occasion, type of diet and allergen-free option, type of cuisine and dish style to plan your meal;<br>2. Cook with step-by-step instructions and videos;<br>3. Make your grocery shop list.	x	x	Gluten and milk (2 food allergens in total).
7. Mercadona	Food products	Android, iOS	1,150	English, +2	1. Search for food products sold in the supermarket and look at the ingredients, allergens and nutritional values on the food product's label;	x	x	Gluten, milk, lactose, crustaceans, fish, mollusks, peanuts, nuts,

					2. Create your shopping list;<br>3. Buy online (only in specific Spanish cities) or search for the supermarket nearest to you;<br>4. Personalized customer service chat			soya, eggs, sesame, mustard, celery, sulfur dioxide and lupin (14 UE allergens + other food allergens).
8. Mi Intolerancia Alimentaria	Food products	Android	1,341	English, +20	1. Set your intolerance and allergy;<br>2. Search a food product to see your compatibility through the colour code alert.	x	x	Gluten, lactose, fructose, histamine, sorbitol and salicylic acid (6 food allergens in total).
9. Open Food Facts	Food products	Android, iOS	1,796	English, + 129	1. Scan the barcode of a product or search for it in the database;<br>2. Look at the ingredients, label, Nutri-score, nutritional values, allergens, instructions on how to recycle the package, traceability, where to buy it and alcohol content for beverages;<br>3. Add new products;<br>4. Set allergen alerts.	x	x	Gluten, milk, lactose, crustaceans, fish, mollusks, peanuts, nuts, soya, eggs, sesame, mustard, celery, sulfur dioxide, lupin, and other allergens present on the food product's label (14 UE allergens + other food allergens).
10. ¿Qué Puedo Comer?	Food products	Android, iOS	1,013	Spanish	1. Make your personal profile and set your allergies/intolerances and preferences (set limit consumption of sugar, salt, saturated fat and palm oil) to find the food product suitable for you;<br>2. Scan the barcode of a product or search for it in the database, and add new ones;<br>3. Find recipes, suggestions and useful documents in the community blog.	x	x	Gluten, milk, lactose, crustaceans, fish, mollusks, peanuts, nuts, soya, eggs, sesame, mustard, celery, sulfur dioxide, lupin and fructose (14 UE allergens + other food allergens).
11. Club VIPS	Restaurants	Android, iOS	12,525	English, +22	1. Set your allergies/intolerances and preferences;<br>2. Select the restaurant near to you and look at the meals suitable for you from the menu;<br>3. Make your order to take away or book a table in a restaurant.	x	x	Gluten, milk, crustaceans, fish, mollusks, peanuts, nuts, soya, eggs, sesame, mustard, celery, sulfur dioxide and lupin (14 UE allergens).
12. Find Me Gluten Free	Restaurants	Android, iOS	8,633	English	1. Search for the gluten-free restaurant near to you and find gluten-free menus from the most popular chain of restaurants;<br>2. Scan food products to share them in the community;<br>3. Look at customers' reviews and ratings;<br>4. Get directions, call and book a table.		x	Gluten (1 food allergen in total).
13. Foster's Hollywood	Restaurants	Android	15,781	Spanish	1. Search for the restaurant near to you;<br>2. Look at the menu and filter by allergens;<br>3. Make your takeaway order or home delivery.	x	x	Gluten, milk, crustaceans, fish, mollusks, peanuts, nuts, soya, eggs, sesame, mustard, celery, sulfur dioxide and lupin (14 UE allergens).

14. Happy Cow	Restaurants	Android	3,357	Spanish	1. Search for vegetarian, vegan and gluten-free restaurants/grocery stores/farmers' market/locals/hotels/caterings/delivery near to you;<br>2. Look at customers' reviews and ratings, and leave your ones;<br>3. Interact with the community.		x	Gluten<br>(1 food allergen in total).
