# Supplementary material for: Mobile Phone Apps for Food Allergies or Intolerances in App Stores: Systematic Search and Quality Assessment Using the Mobile App Rating Scale (MARS)
Source: JMIR Mhealth Uhealth. 2020 Sep 16;8(9):e18339. doi: 10.2196/18339 (PMC7527917; doi:10.2196/18339)
Supplement: Multimedia Appendix 2 [file mhealth_v8i9e18339_app2.pdf]

		Meal planner apps						Food product apps				Restaurant apps			
	Total n (%)	Eat This Much	Fitberry	Mealime	Recetas Vegetarianas y Veganas	Side Chef	Tasty	Mercadona	Mi Intolerancia Alimentaria	Open Food Facts	¿Qué Puedo Comer?	Club VIPS	Find Me Gluten Free	Foster's Hollywood	Happy Cow
**Search filter**	13/14 (93)	x	x	x	x	x	x	x	x		x	x	x	x	x
**Restaurant/ store finder**	5/14 (36)							x				x	x	x	x
**Self-monitoring and feedback**	2/14 (14)	x[a]		x											
**Reminders and notification**	8/14 (57)	x	x	x	x	x	x			x	x				
**Incentives**	0/14 (0)														
**Shopping list**	10/14 (71)	x[a]	x	x	x	x	x	x				x		x	x
**Remote support**	9/14 (64)	x	x	x		x	x				x		x	x	x
**Prompts and discounts**	7/14 (50)		x					x			x	x	x	x	x
**Barcode scanner**	4/14 (29)									x	x	x	x		
**Nutritional info**	8/14 (57)	x		x[a]	x	x	x	x		x	x				
**Allergens detection**	14/14 (100)	x	x	x	x	x	x	x	x	x	x	x	x	x	x

**Ingredients and additives list**	11/14 (79)	x	x	x	x	x	x	x	x	x	x			x	
**Suggestions and tips**	8/14 (57)	x	x	x	x	x	x				x				x
**Food products info**	8/14 (57)	x			x	x		x		x	x		x	x	
**Food recipes**	8/14 (57)	x	x	x	x	x	x				x				x
**Geolocalization**	6/14 (43)							x			x	x	x	x	x
**Registration**	5/14 (36)	x				x		x			x		x		
**Paid version (Premium)**	6/14 (43)	x	x	x					x				x		x
**Data back-up**	4/14 (29)	x		x						x					x
**Web-based version**	9/14 (64)	x		x		x				x	x	x	x	x	x

[a]: Only in the paid premium version.
