# Supplementary material for: Mobile Phone Apps for Food Allergies or Intolerances in App Stores: Systematic Search and Quality Assessment Using the Mobile App Rating Scale (MARS)
Source: JMIR Mhealth Uhealth. 2020 Sep 16;8(9):e18339. doi: 10.2196/18339 (PMC7527917; doi:10.2196/18339)
Supplement: Multimedia Appendix 3 [file mhealth_v8i9e18339_app3.pdf]

		Meal planner apps						Food product apps				Restaurant apps			
	Total n (%)	Eat This Much	Fitberry	Mealime	Recetas Vegetarianas y Veganas	Side Chef	Tasty	Mercadona	Mi Intolerancia Alimentaria	Open Food Facts	¿Qué Puedo Comer ?	Club VIPS	Find Me Gluten Free	Foster's Hollywood	Happy Cow
**Personal profile**	14/14 (100)	x	x	x	x	x	x	x	x	x	x	x	x	x	x
**Favourite list**	11/14 (79)	x	x	x	x	x	x		x		x	x	x		x
**Online purchase**	9/14 (64)		x	x		x		x	x		x	x	x[a]	x	
**Rate/review**	14/14 (100)	x	x	x	x	x	x	x	x	x	x	x	x	x	x
**Social sharing and community**	13/14 (93)	x	x	x	x	x	x		x	x	x	x	x	x	x
**Meal planning**	7/14 (50)	x	x	x	x	x	x							x	
**Set daily goals**	4/14 (29)	x		x		x	x								
**Edit and add info, products and notes**	10/14 (71)	x	x	x	x	x			x	x	x		x		x
**Connection with other devices (Health app)**	2/14 (14)	x		x											

[a]: Premium version only.
