# Supplementary material for: Mobile Phone Apps for Food Allergies or Intolerances in App Stores: Systematic Search and Quality Assessment Using the Mobile App Rating Scale (MARS)
Source: JMIR Mhealth Uhealth. 2020 Sep 16;8(9):e18339. doi: 10.2196/18339 (PMC7527917; doi:10.2196/18339)
Supplement: Multimedia Appendix 4 [file mhealth_v8i9e18339_app4.pdf]

App name (purpose)	MARS app objective quality[a]					MARS app subjective quality[a]	MARS app-specific[a]	App store user star ratings[b]	MARS app classification				
	Sections								Items				
	Engagement	Functionality	Esthetics	Information	Total[c]				Focus	Theoretical strategies	Affiliation	Age group	Technical aspects
Eat This Much (meal planners)	4.4 (0.5)	5.0 (0)	4.3 (0.6)	4.3 (0.8)	4.5 (0.3)	4.5 (0.6)	4.5 (0.5)	4.5	Increase well-being; behavior change; other: healthcare.	Monitoring /tracking; goal setting; advice/tips.	Commercial	General	Sharing; password protection; login; reminders; needs web access.
Fitberry (meal planners)	3.4 (0.9)	3.2 (0.5)	4.0 (0)	3.5 (0.5)	3.5 (0.3)	3.2 (0.5)	3.0 (0)	4.3	Increase well-being; behavior change; other: healthcare.	Info /education; advice/tips.	Commercial	General	Sharing; password-protection; reminders; needs web access.
Mealime (meal planners)	4.4 (0.5)	5.0 (0)	4.7 (0.6)	4.0 (0.6)	4.5 (0.4)	4.5 (0.6)	4.0 (0)	4.5	Increase well-being; behavior change; other: healthcare.	Info/ education; monitoring /tracking; goal setting; advice/tips.	Commercial	General	Sharing; community; password protection; reminders; needs web access.
Recetas Vegetarianas y Veganas (meal planners)	4.2 (0.8)	3.7 (0.5)	4.0 (0)	4.0 (0.6)	4.0 (0.2)	3.5 (0.6)	4.3 (0.5)	4.6	Increase well-being; behavior change; other: healthcare.	Info /education; advice/tips.	Commercial	General	Sharing; reminders; needs web access.
SideChef (meal planners)	4.2 (0.4)	4.7 (0.5)	4.7 (0.6)	4.2 (1.0)	4.4 (0.3)	4.0 (0.8)	4.0 (0)	4.3	Behavior change; other: healthcare.	Info /education; goal setting; advice/ tips.	Commercial	Young adult; adult.	Sharing; password-protection; login; needs web access.
Tasty (meal	4.0 (1.0)	4.0 (0)	4.0 (0)	3.8 (0.7)	3.9 (0.1)	3.5 (0.6)	4.0 (0)	4.7	Increase well-being;	Info /education;	Commercial	General	Sharing; reminders;

planners)									behavior change; other: healthcare.	monitoring /tracking; advice/tips.			needs web access; needs web access.
Mercadona (food products)	2.6 (0.5)	4.0 (0)	3.3 (0.6)	3.5 (0.5)	3.3 (0.6)	2.5 (1.0)	3.0 (0)	3.1	Increase well-being; other: healthcare.	Info /education; advice/tips.	Commercial	General	Needs web access.
Mi Intolerancia Alimentaria (food products)	2.6 (0.9)	3.7 (0.5)	3.3 (0.6)	3.2 (0.7)	3.2 (0.5)	3.0 (0)	3.2 (0.4)	4.1	Increase well-being; other: healthcare.	Info /education	Commercial	General	Sharing; needs web access.
Open Food Facts (food products)	3.0 (1.2)	5.0 (0)	3.3 (0.6)	4.5 (0.5)	3.9 (0.9)	4.2 (0.9)	3.7 (0.5)	4	Other: increase awareness, supply info and healthcare.	Info/ education	NGO	General	Community; password protection; needs web access.
¿Qué Puedo Comer? (food products)	3.8 (0.4)	3.7 (0.5)	4.7 (0.6)	4.0 (0.6)	4.0 (0.4)	3.5 (0.6)	4.0 (0)	4	Other: increase awareness, supply info and healthcare.	Info /education; advice/tips.	Commercial	General	Sharing; community; password protection; login; reminders; needs web access.
Club VIPS (restaurants)	3.2 (0.8)	3.5 (0.6)	3.3 (0.6)	3.8 (0.4)	3.5 (0.3)	2.7 (0.5)	3.0 (0)	3.8	Increase well-being; other: healthcare.	Info /education	Commercial	Young adult; adult.	Password protection; needs web access.
Find Me Gluten Free (restaurants)	3.0 (1.0)	4.0 (0)	4.0 (0)	3.8 (0.7)	3.7 (0.5)	3.5 (0.6)	4.0 (0)	4.5	Increase well-being; other: healthcare.	Info /education	Commercial	General	Sharing; password-protection; login; needs web access.
Foster's Hollywood (restaurants)	3.0 (0.7)	3.5 (0.6)	4.0 (0)	2.8 (0.7)	3.3 (0.5)	3.0 (0)	2.0 (0)	3.7	Increase well-being; entertain.	Info /education	Commercial	General	Sharing; community; needs web access.

Happy Cow (restaurants)	3.6 (0.5)	3.7 (0.5)	4.0 (0)	4.0 (0.6)	3.8 (0.2)	3.5 (0.6)	4.0 (0)	4.4	Increase well-being; other: healthcare; entertain.	Info /education; advice/tips.	NGO	Young adult; adult.	Sharing; community; password protection; needs web access.

NGO: Non-Governmental Organization;

[a]: This section is rated from 0 to 5 points in mean scores (SD);

[b]: User star ratings extracted from app stores, intended as the average rating given by the users to the app from a scale of 1 to 5 stars;

[c] :Total score of engagement + functionality + esthetics + information sections.
